# Assessing the applicability of public health intervention evaluations from one setting to another: a methodological study of the usability and usefulness of assessment tools and frameworks

**DOI:** 10.1186/s12961-018-0364-3

**Published:** 2018-09-04

**Authors:** Helen Elizabeth Denise Burchett, Laurence Blanchard, Dylan Kneale, James Thomas

**Affiliations:** 1 Faculty of Public Health & Policy, London School of Hygiene & Tropical Medicine, 15-17 Tavistock Place, London, United Kingdom; 2 Evidence for Policy and Practice Information and Coordinating Centre, UCL Institute of Education, University College London, London, United Kingdom

**Keywords:** Applicability, Generalisability, External validity, Transferability

## Abstract

**Background:**

Public health interventions can be complicated, complex and context dependent, making the assessment of applicability challenging. Nevertheless, for them to be of use beyond the original study setting, they need to be generalisable to other settings and, crucially, research users need to be able to identify to which contexts it may be applicable. There are many tools with set criteria for assessing generalisability/applicability, yet few seem to be widely used and there is no consensus on which should be used, or when. This methodological study aimed to test these tools to assess how easy they were to use and how useful they appeared to be.

**Methods:**

We identified tools from an existing review and an update of its search. References were screened on pre-specified criteria. Included tools were tested by using them to assess the applicability of a Swedish weight management intervention to the English context. Researcher assessments and reflections on the usability and utility of the tools were gathered using a standard pro-forma.

**Results:**

Eleven tools were included. Their length, content, style and time required to complete varied. No tool was considered ideal for assessing applicability. Their limitations included unrealistic criteria (requiring unavailable information), a focus on implementation to the neglect of transferability (i.e. little focus on potential effectiveness in the new setting), overly broad criteria (associated with low reliability), and a lack of an explicit focus on how interventions worked (i.e. their mechanisms of action).

**Conclusion:**

Tools presenting criteria ready to be used may not be the best method for applicability assessments. They are likely to be either too long or incomplete, too focused on differences and fail to address elements that matter for the specific topic of interest. It is time to progress from developing lists of set criteria that are not widely used in the literature, to creating a new approach to applicability assessment. Focusing on mechanisms of action, rather than solely on characteristics, could be a useful approach, and one that remains underutilised in current tools. New approaches to assessing generalisability that evolve away from checklist style assessments need to be developed, tested, reported and discussed.

**Electronic supplementary material:**

The online version of this article (10.1186/s12961-018-0364-3) contains supplementary material, which is available to authorized users.

## Background

Public health interventions can be complicated, complex and context dependent [1–3]. This makes consideration of whether a public health intervention is suitable for other contexts challenging. Nevertheless, for an intervention to be of use beyond the setting in which it was originally evaluated, it needs to be generalisable to other settings and, crucially, research users need to be able to identify which contexts it may be applicable to.

Interest in generalisability (i.e. to which unspecified settings a study’s findings could be generalised) and applicability (i.e. the likelihood that an intervention could be applied to a new, specific setting) has increased in recent years, at least in terms of the number of publications discussing these issues. There have been calls for greater attention to generalisability or applicability [4–7], with concerns about the lack of guidance offered [8–10] and many papers noting the insufficient reporting of relevant information for their assessment [10–20]. Reporting guidelines for randomised trials, non-randomised trials, observational studies and qualitative research all include consideration of generalisability (or relevance) [21–25]. However, although they may offer examples of criteria to consider, none offer a detailed explanation of how to assess whether findings (of either primary studies or systematic reviews) are generalisable to another context.

Methodological progress does not appear to have kept pace with this contemporary interest. A review published in 2011 looked at the features of frameworks and tools for assessing generalisability/applicability (since a range of terms are used, hereafter we will refer to all as ‘tools’) [26]. Since then, new tools have been published [27–31], as well as new reviews of tools [8, 29, 32, 33]. Despite this proliferation of tools, there remains a notable absence of consensus in the published literature on the appropriate method for the assessment of applicability, and few tools appear to be used widely.

Assessing the applicability of interventions is not only useful for primary research and programme implementation, systematic reviewers also need to consider applicability in order to better meet decision-makers’ needs [8, 34–37]. In an attempt to encourage the field to move beyond a recurring cycle of tool development without subsequent use, we conducted a methodological study aiming to test existing published tools. This study intends to reflect on how easy they were to use and how useful they appeared to be in assessing the applicability of a public health intervention to a different context.

## Methods

To be objective and transparent in the identification of tools to be tested, although this was not a review, systematic search principles were used. Tools were identified from an existing, broader systematic review and that review’s search (of five databases) was updated to December 31, 2017 (see earlier review for details of search strategy) [26]. Additional relevant papers were sought from the reference lists of the 25 tools identified in the previous review and newly included papers.

Papers were screened, initially on title and abstract and, if included at that stage, on full text. Papers were excluded if they (1) were not in English, (2) did not explicitly aim to present a means of assessing applicability (e.g. presented criteria for describing applicability rather than evaluating it), (3) did not present a clear set of criteria to be used by others (e.g. the criteria were not ready to be used, or were not easily identifiable as part of a list or in a text), (4) included criteria on questions broader than applicability (e.g. decision-making, or other aspects of evidence appraisal, e.g. internal validity), (5) focused on economic evaluations, (6) were not multi-domain (e.g. included criteria related to population alone and not broader conceptualisations of applicability), or (7) focused on decision-making at the individual (e.g. patient) level.

In order to assess their usability and usefulness, each included tool was used in an applicability assessment and the experience of using it was recorded. Each tool was used to assess the applicability of a Swedish weight management intervention by Bertz et al. [38–41] to the English context (the resources and practices in England in general). Although any intervention evaluation could have been used to test the tools, this study was chosen for two reasons. Firstly, it had been identified as highly effective in a recent review [42] (there is arguably little to be gained from assessing the applicability of ineffective interventions) and, secondly, because it included a qualitative process evaluation. It was expected that this qualitative component would offer insights into the context, implementation and experience of the intervention, which would provide useful information for the applicability assessments. The intervention consisted of four arms and the assessment of generalisability was focused on the dietary behaviour modification group.

To complete the applicability assessment, information was obtained about the study context (e.g. population characteristics, material resources, health behaviours) from the study’s publications, with supplementary data for Sweden and equivalent data for England sourced from simple internet searches or from the existing knowledge and experience of the person using the tool. Although more detailed and thorough information searches could have been conducted, it was felt that these would require an excessive amount of time and resources to ensure accuracy but would add little to our understanding of the tools’ usability and utility.

Each tool was tested by one of three researchers (HB, LB, DK), except for one [43], which was tested by two (LB, DK), in order to compare their interpretations and experiences directly and explore the degree of subjectivity of assessments. To record the experiences using the tools, a standard pro-forma was completed to record researchers’ reflections on each criterion and the tool as a whole (e.g. how easy it had been to use, which criteria were or were not considered useful and, based on that specific tool, how applicable the intervention was felt to be to the English context). An example of how this was completed can be found in Additional file 1. Further, the three researchers met regularly during the testing period to reflect on their experiences.

## Results

The search update identified 3380 references, of which 1109 were duplicates, leaving 2271 references to be screened, in addition to those from the earlier review. Eleven tools were included (see Table 1 for details) [27–30, 34, 35, 37, 43–46].

**Table 1:** Characteristics of included tools

Reference	Aim/Description of tool	Criteria	Summary of sub-criteria^a^	Example criterion
Atkins et al. (2011) [34]	Factors organised by the PICOS framework, which may limit the applicability of individual studies	1. Population	1. Eligibility criteria; differences in demographics, representativeness, exclusion rate of participants; unrepresentative event rates	“*Large differences between demographics of study population and community patients*”, p. 1201
		2. Intervention	2. Unrepresentative of current practice; not feasible; comparison with current standard; co-interventions; unrepresentative providers or provider training	
		3. Comparator	3. Inadequate dose; substandard alternative treatment	
		4. Outcomes	4. Composite outcomes; short-term/surrogate outcomes	
		5. Setting	5. Different standards of care; unrepresentative population or level of care	
Bonell et al. (2006) [43]	Framework for empirically assessing and reporting generalisability of randomised trials	1. Can the intervention be delivered elsewhere?	1. Feasibility; coverage; acceptability	“*Secondly, an intervention must achieve adequate coverage. This may depend on the overall comprehensiveness of health systems or on whether providers can reach people in other ways—for example, through outreach. Adequate coverage may be more difficult in some sites or sub-populations*”, p. 346
		2. Does the intervention meet recipients’ needs?	2. Similar needs	
Burford et al. (2013) [27]	Questions to guide the assessment of the applicability of the findings of a systematic review to a specific setting	1. Studies conducted in same setting/findings consistent across settings/times?		“*Are there any political, social, or cultural factors that may affect the implementation of this intervention?*”, p. 1259
		2. Are there important differences in on-the-ground realities and constraints that might substantially alter the feasibility and acceptability of an option?	2. Political, social or cultural factors that may affect implementation; acceptability to general public; ethically acceptable; does target population have sufficient means to receive/implement intervention; can intervention be tailored?	
		3. Are there important differences in health system arrangements that may mean an option could not work in the same way?	3. Organisation responsible for intervention and organisational structure as a barrier to implementation; capacity to implement	
		4. Are there important differences in the baseline conditions that might yield different absolute effects even if the relative effectiveness was the same?	4. Baseline prevalence; population characteristics	
		5. What insights can be drawn about options, implementation, and monitoring and evaluations?	5. Sufficient resources; skills/training for providers	
Cambon et al. (2013) [28]	Tool to analyse transferability and to support the development and adaptation of health promotion interventions (primary research) to new settings	1. Population	1. Epidemiologic and sociodemographic characteristics; cognitive, cultural, social and educational characteristics; motivation; accessibility; climate of trust between providers and recipients; recipient population’s belief in the intervention’s utility; recipient population’s demand for intervention; recipient population’s perceptions of their health needs; acceptability to recipient population; participation levels; degree of involvement	“*The epidemiologic and sociodemographic characteristics of the recipient population are similar in the primary and replica interventions Subcriteria: Socioeconomic characteristics (rate of unemployment); demographic characteristics (age, sex); health status*”, p. 12, Supplementary file 2
		2. Environment	2. Supportive institutional environment; supportive other elements of context; partnerships	
		3. Implementation	3. Intervention methods; resources available; skills of providers and project leader; providers’ belief in intervention’s utility; acceptability to providers; mobilisation of providers	
		4. Support for transfer	4. Adaptations; transfer elements prepared and provided; knowledge transfer process	
Green & Glasgow (2006) [44]	Evaluation questions related to the RE-AIM dimensions to aid the planning, conduct, evaluation and reporting of studies having the goal of translating research into practice	1. Reach (individual level)	1. Participation rate and representativeness	“*Participation rate among intended audience and representativeness of these participants. Questions to ask: What percentage of the target population came into contact with or began program? Did program reach those most in need? Were participants representative of your practice setting?*”, p. 133
		2. Effectiveness (individual level)	2. Impact on key outcomes and quality of life; consistency of effects across subgroups	
		3. Adoption (setting and/or organisational level)	3. Participation rate and representativeness of settings	
		4. Implementation (setting and/or organisational level)	4. Level and consistency of delivery	
		5. Maintenance (individual and setting levels)	5. Long-term effectiveness (individual level); sustainability and adaptation (setting level)	
Gruen et al. (2005) [35]	Questions to assess the generalisability of findings of systematic reviews	1. Relative importance of the health problem	1. Occurrence and severity of health problem	“*Relative importance of the health problem: do the occurrence and severity of the health problem vary significantly between settings and how might this affect the intervention’s potential benefit to the population?*”, p. 480
		2. Relevance of outcome measures	2. Meaningfulness of outcome measures	
		3. Practicality of the intervention	3. Factors that may affect feasibility	
		4. Appropriateness of the intervention	4. Are other interventions more appropriate	
		5. Cost-effectiveness of the intervention	5. Costs and benefits	
Khorsan & Crawford (2014) [29]	Tool to assess the external validity of randomised controlled trials and non-randomized studies in healthcare interventions	1. Recruitment	1. Identification and recruitment	“*Recruitment: Did the study identify the source population for participants and describe how the participants were recruited from that source population?*”, p. 8
		2. Participation	2. Representativeness	
		3. Model validity	3. Representativeness of staff, places and facilities	
Lavis et al. (2004) [45]	Approach to assess the local applicability of systematic reviews of health systems research	1. Could it work?	1. Structural elements of the health system	“*Could it work? Are there important differences in the structural elements of health systems (or health system subsectors such as pharmaceuticals or home care) that mean an intervention could not work in the same way as in the countries where the research was done – e.g., institutional attributes such as the degree of integration in service delivery*”, p. 1618
		2. Will it work? (or what would it take to make it work?)	2. Perspectives and influence of health system stakeholders; other health system challenges; power dynamics and on-the-ground realities and constraints (and ability to change these)	
		3. Is it worth it?	3. Balance of benefits and harms	
Lavis et al. (2009) [37]	Questions to guide the assessment of the applicability of a systematic review’s findings to a specific setting	1. Were the studies included in a systematic review conducted in the same setting or were the findings consistent across settings or time periods? 2. Are there important differences in on-the-ground realities and constraints that might substantially alter the feasibility and acceptability of an option? 3. Are there important differences in health system arrangements that may mean an option could not work in the same way? 4. Are there important differences in the baseline conditions that might yield different absolute effects even if the relative effectiveness was the same? 5. What insights can be drawn about options, implementation, and monitoring and evaluation?		“*Are there important differences in on-the-ground realities and constraints that might substantially alter the feasibility and acceptability of an option?*”, p. 4
Schoenwald & Hoagwood (2001) [46]	Dimensions and variables that can be used to compare conditions in research settings and practice settings	1. Intervention characteristics	1. Nature of treatment theory; focus of treatment; specification of treatment; similarity of new and prevailing treatment; complexity and clarity of intervention model	“*Intervention characteristics: Nature of the treatment theory, including the relative weight of the theoretical, empirical, and clinical base; focus of the treatment: specific vs diffuse; specification of the treatment, including whether manuals are used and how comprehensive and prescriptive they are; similarity of the treatment to the prevailing practice for treating the identified problem or problems; complexity of the intervention model; clarity of the intervention model*” p. 1194
		2. Practitioner characteristics	2. Specialised training; adherence monitoring; clinical supervision, supervisor; type of practitioner; endorsement of intervention model; salary; anticipated job longevity	
		3. Client characteristics	3. Referral problem; family context; referral source; age, gender, ethnicity	
		4. Service delivery characteristics	4. Frequency, length and location of sessions; source of payment for service	
		5. Organisational characteristics	5. Organisational structure including hierarchy; personnel policies; organisational culture and climate; size; mission; mandate	
		6. Service system characteristics	6. Policies and practices of referral/payers; financing; legal mandates; interagency working relationships	
Young & Borland (2011) [30]	Five dimensions to consider in order to generalise knowledge to practice from any given corpus of research	1. The nature of the problem or issue being intervened in 2. The characteristics of the population that is the target of the intervention 3. The context of the intervention 4. The nature of the intervention mechanism itself 5. Framework (the formal or informal set of beliefs that frame the intervention)		“*The nature of the problem or issue being intervened in: The task here is to map those characteristics of the problem that are relevant to the choice of intervention. There are two dimensions to this; variation of the focal behaviour around whatever is normative and the focal behaviour’s relationship with conceptually related behaviours*”, p. 263

^a^ For exact wording, see original article

### Tool characteristics

All 11 included tools were generic, i.e. they were not designed for use with a specific topic or setting. Most aimed to assess intervention evaluations individually, although three aimed to assess the applicability of systematic review findings [27, 37, 45].

The tools varied widely in terms of their length, content and style. Some were long and detailed, with more than 20 questions and with templates provided for use (e.g. [28]). Others contained fewer questions which were broader, supported by examples of the types of factors to consider when answering them (e.g. [43, 45]). However, guidance on how to use the tools, or what information to draw on, was generally limited across all tools.

Initially, when we considered the tools, before attempting to apply them, many appeared to be useful. However, it was only when we began to apply the tools to a specific intervention evaluation and context that we realised how challenging their use was and that they may not actually be as useful as we had initially thought.

We now consider the usability of the tools and then their utility in terms of the aspects of applicability assessed, their validity and their reliability.

### Usability

The amount of time required to complete the tools varied from relatively short (under half an hour in some cases) (e.g. [29, 35, 37, 45]) to those taking a long time (over 3 hours in some cases) (e.g. [28, 30, 46]). The time taken also varied by researcher, depending on how much detail was considered necessary to address the criteria. This was found to be quite subjective; indeed, the extent of and time for searches varied depending on the questions asked in the tools, information available in the papers, previous knowledge of the researchers (especially of the English context), the level of depth with which they felt confident and the amount of time they were ready to invest. Had we attempted to search for and appraise data sources (beyond minimal internet searches), more time would be required. However, there did not appear to be a link between the time taken and the perceived utility of the tools. Indeed, no tool seemed ideal for assessing applicability.

Some criteria did not seem realistic to answer, since information would be unlikely to be available either from the original study reporting (unless a comprehensive process evaluation was conducted and published) or in the proposed new context. For example, the ASTAIRE tool by Cambon et al. asks whether “*the climate of trust between providers and recipients is similar in the primary and replica interventions*” ([28], p. 9S). Whilst the climate of trust in the study context may (rarely) have been reported in a process evaluation, in the proposed new context, this information would likely only be available as implicit knowledge among those familiar with the context, rather than in a written format. Furthermore, factors such as trust may vary within a context, depending on how an intervention is implemented. It would be difficult, if not impossible, to make a judgement about it at this stage, although consideration could help to shape the implementation process. This example also illustrates that the purpose of the tools was not always made explicit and, consequently, they often swayed between tools for the assessment of evidence and aids for implementation.

Some questions in the tools could only be answered accurately by decision-makers themselves, rather than researchers, e.g. questions about costs. A high cost does not automatically imply that an intervention is not feasible; it depends on the overall budget and the perceived value of the intervention and competing priorities.

### Utility – aspects of applicability

Certain aspects that could be important for applicability assessments were frequently neglected. All tools placed a greater focus on the likelihood of replicating the implementation of the intervention than on replication of the intervention effects. In several tools, it was not clear whether the transferability of the intervention’s original effectiveness was being considered [29, 30, 43, 46]. For example, criteria concerning population characteristics could affect the applicability of implementation or the transferability of effects, or both. Frequently, the expected focus was not made explicit to the user; for example, criteria focusing on whether or how an intervention could ‘work’ were often ambiguous [27, 30, 37, 45], since ‘work’ could mean either implementation or effectiveness. In addition, the tools focused on intervention delivery to the neglect of other aspects of the intervention process such as the applicability of the recruitment method, or whether and how interventions and their effects changed over time.

### Utility of tools – validity

Tools did not always steer users to focus specifically on those characteristics known to impact on applicability. For example, Burford et al.’s tool asked “*Are the characteristics of the target population comparable between the study setting(s) and the local setting?*” ([27], p. 1259). In the Bertz study [38], women were eligible for inclusion if they intended to breastfeed for 6 months, and all included participants were found to be breastfeeding, almost all exclusively. In England, breastfeeding rates were lower than this, at 43% at 6–8 weeks postpartum in 2015/2016 [47]; exclusive breastfeeding at 6 weeks was 24% in 2010 [48]. This may not affect the applicability of the intervention implementation to the English context, but could affect the transferability given that there is evidence that breastfeeding is associated with greater weight loss [49]. This may mean a smaller effect size may be found in an English population, even if implementation remains identical.

The women included in the Bertz intervention were also found to be substantially older (mean age, 33.7 years in the diet group) than the mean age at motherhood (regardless of parity) of mothers in both Sweden (31.0 years in 2014) and the United Kingdom (30.2 years) [50]. In contrast to the example of breastfeeding above, age is not found to be associated with postpartum weight loss and therefore may not need to be considered in this particular applicability assessment [51]. The absence of focus in the criteria, with no accompanying guidance, encourages data-driven assessments of generalisability. Had the user focussed on differences in age between the populations alone and not breastfeeding, summary judgements about the transferability of the evidence may have been made that were erroneous. Identifying which factors that may influence its applicability could lead to more accurate assessments, rather than relying on fixed, potentially irrelevant, ‘standard’ factors such as age, sex, income and educational level.

Only one tool explicitly considered “*the nature of the intervention mechanism itself*” ([30], p. 264), and another considered it within a criterion about adaptation: “*Adaptations can be (or were able to be) made to the primary intervention in the replica context without altering its fundamental nature*” ([28] p. 14, S2). However, an understanding of the underlying mechanisms seemed essential in order to appropriately apply a number of the tools’ criteria, particularly in terms of considering adaptations. For example, there are likely to be a range of ways to consider, “*can the intervention be tailored to suit the implementation setting?*” ([27], p. 1259). The frequency, duration or location of sessions could be altered, different providers could be used or different messages could be given to participants. All of these factors could be changed, or just one of them. However, the critical point is that these adaptations should not affect the way the intervention exerts its effect – so that the mechanism of action, and ultimately the outcome, is not altered. For example, in the Bertz study, dieticians were used to deliver the intervention [38]. In the United Kingdom, the British Dietetic Association has stated that there are “*insufficient dietitians in the UK to meet current needs, let alone the much wider roles that we believe they could perform*” ([52], p. 2), suggesting that either training and employing many more dietitians, or using alternative providers, would be necessary for scaling up the intervention in England. The study’s process evaluation highlighted the importance participants’ placed on providers’ “*professional credibility*” ([39], p. 637), so it would be important to understand whether participants in England would also perceive other providers to have professional credibility, otherwise the intervention’s effect may not be replicated.

### Utility – reliability

Four tools included questions so broad they required consideration of multiple factors simultaneously (which was not always clearly stated) [29, 37, 43, 45]. Broad questions were open to different interpretations, e.g. “*Are there important differences in on-the-ground realities and constraints that might substantially alter the feasibility and acceptability of an option?*” ([37], p. 4). The tool user could focus on different elements here, such as provider workload, the number or type of providers available, provider motivation levels, the location of services, attitudes, existing practices and so on. In practice, it would be simpler for a tool user to focus on elements for which information was available, or for which a judgement was easier to make; however, without further guidance, this approach could lead to the omission of those aspects most pertinent to applicability. For example, information about the number of providers in the study setting and new setting may be more easily available than information about providers’ motivation to deliver the intervention, yet the latter may be equally or more important for applicability assessments.

These broad criteria could result in assessments remaining as implicit and potentially incomplete as those made without a tool. Broader criteria increase the risk that the user’s background knowledge, experience and interests influence their judgments. This was confirmed by the two assessments undertaken (by LB and DK) using the same tool [43]. For example, the first of the tool’s four questions focused on feasibility; “*can the intervention be delivered elsewhere*” ([43], p. 346). One researcher answered this question as, ‘possibly’, focusing on the intervention approach and feasibility of the referral system and other factors. However, the other researcher felt that it was unlikely to be feasible at scale in England due to the lower number of dietitians available in the United Kingdom to deliver the intervention, because home visits may not be possible in rural areas, and due to the relatively high costs. The wide range of factors encompassed within ‘feasibility’, from costs to providers, referral systems and settings, mean that it is up to the tool user to decide which specific aspects to focus on and prioritise. Although it could be argued that this is inevitable for generic tools, it seems likely that topic-specific tools would face a similar challenge, since there may still be a wide range of interventions and contexts within a single topic. Furthermore, the inevitability of this weakness does throw into question the reliability and purpose of an approach to assessing applicability using a generic tool, particularly when that tool is not supported by guidance on how to use it. Out of the 11 tools included, only six offered instructions or guidance and/or a concrete example on how to use the tool [28, 29, 34, 35, 37, 43]. However, all instructions were limited and no example showed how to compare the applicability of one context to another (they all consisted of description of studies only).

The lack of guidance, combined with the breadth of some criteria and their subjective nature, led to different interpretations of the applicability of the illustrative study to the English context. Summary judgements varied depending on the focus of the tool and the user. Most tools led the user to judge the intervention as not, or possibly not, applicable. A minority of tools (3 out of 11) supported a judgement that the evidence had reasonable applicability to England, albeit with caveats [30, 35, 45]. Common characteristics of these three tools were that they were less structured, necessitated a degree of flexibility of interpretation and generally sought out high-level conceptual judgements, as opposed to considering more detailed information about the intervention delivery. Unlike the other 10 tools, 1 of these 3 included a strong focus on exploring the mechanisms of action [30].

In contrast, tools that focussed on obtaining and contrasting more detailed information about aspects of feasibility, such as intervention provider characteristics, generally led to judgements that the evidence and intervention were not applicable to the English context [27–29, 34, 43, 44, 46]. Only two tools directly encouraged the consideration of modifications to the delivery of the intervention that could overcome barriers to implementation [27, 28]. Several of the tools that led users to judge the evidence as inapplicable focused on differences between settings or populations rather than considering both similarities and differences.

## Discussion

Overall, the tools covered a wide range and large number of criteria. Their use and interpretation varied between users in terms of the time taken, level of detail sought, focus and overall judgments made. However, no tool was felt to be ideal for the assessment of applicability, either in terms of usability or utility.

We believe that tools with set criteria are not the ideal way to assess applicability for four main reasons. First, a standardised list of criteria is unlikely to be useful or usable. Combining all the criteria considered ‘useful’ from all the tools into one new tool would create a long list of criteria, requiring an unfeasible amount of time to complete. The Medical Research Council guidance on process evaluations defined context as including, “*anything external to the intervention that may act as a barrier or facilitator to its implementation, or its effects*” ([53], p. 2). There is therefore a potentially limitless number of factors that could be considered in an applicability assessment, but only those factors that may affect the implementation and effectiveness should be considered.

Second, the criteria pertinent to an assessment of applicability will vary depending on the specific topic. In the example given above, breastfeeding seems a relevant population characteristic, whereas for another topic a different population characteristic may be relevant. Although it could be argued that tools should be a prompt for people to consider what issues are most important within each overall criterion, none helped the user to ascertain which issues, for their specific topic, should be considered. It seemed that the tools implicitly assumed that the user held a deep understanding of how the intervention worked, so that they were able to focus only on those specific aspects of the criteria that were pertinent. However, we believe that this is often not the case. We argue that, if having this a priori understanding is a prerequisite, it needs to become much more explicit and, relatedly, that primary studies should focus more on evaluating and reporting how an intervention worked.

Third, it is now well recognised that the context and process of intervention implementation, as well as the intervention design itself, are important factors influencing outcomes [53–55]. Yet, no existing tool directly steered users to critically explore the interrelationship between intervention design, implementation and setting. Therefore, many of the key mechanisms and elements that could matter for an intervention to be applicable to new settings were left unidentified or were not considered. If it were possible to observe an intervention’s effect numerous times, in identical contexts, an understanding of the mechanisms of action may not be necessary to ascertain that the intervention is effective. Such is the case for pharmaceutical studies. However, it is rare for a public health intervention to be perfectly replicated, without any adaptation or changes to implementation or content, in identical contexts. Therefore, only by understanding the mechanisms of action through which an intervention exerts its effect – and which contextual elements underpin them – can we know what an assessment of applicability should focus on. By focusing on the mechanisms of action, we focus on how the intervention works and its potential interactions with context, rather than differences in characteristics of the population, intervention or context. Once the mechanism of action is understood, the specific criteria to consider in assessing applicability should become clearer. Whether aspects of the intervention could or should need adaptation in order to enable the replication of the mechanism of action, can also then be considered.

Fourth, checklist-style tools lean towards conceptualising applicability as a binary concept – is it applicable or not? With such a closed question, it is far easier to conclude that an intervention is not applicable rather than that it is – it is easier to identify some or any differences, than to identify sufficient similarities for applicability (who is able to judge what is sufficiently similar?). At this point, it is useful to think about the purpose of an applicability assessment. For example, in a systematic review, are assessments conducted in order to identify which studies should be included or excluded? Or how studies should be weighted within a review? We believe the utility of applicability assessments could go beyond these, but concepts of applicability need to shift to a more multifaceted view, recognising that it is a multi-domain construct. Applicability assessments could then help to answer the questions of how could an intervention’s mechanisms of action be replicated or which issues are important to consider. The questions then focus more on what could be learnt from this study, even if the context in the new setting is different, or if the intervention cannot be delivered exactly as it was in the original setting.

We are not the first to recognise the limitations of tools with set criteria. For tools assessing the quality of qualitative research, Barbour pointed out the concern that they may be used “*prescriptively*” and in an “*uncritical*” way ([56], p. 1115), inhibiting a deeper and broader understanding. Nor is a focus on mechanisms of action a new concept per se – others have highlighted the importance of programme theories or causal pathways for understanding and evaluating the effectiveness of interventions [53, 57–59]. We argue that not only is it needed for understanding intervention evaluations, it is also necessary for assessments of applicability.

This study is not without its limitations. Firstly, only English-language tools were included and tools that had a broader focus than applicability (e.g. considered internal validity as well) were excluded. However, we believe that the overall conclusion of the paper would not have been affected had they been included, since such tools contain similar checklists of criteria rather than different approaches to assessing applicability. All but one of the tools were applied by only one researcher, with limited time and resources used to identify relevant contextual information. However, in order to explore and highlight the issue of subjectivity, one tool was applied by two researchers and compared. Furthermore, we believe it is likely that, if tools were to be used, it would often be by individuals rather than groups of people. Although more time could have been spent collecting contextual data, given the challenges of using the tools, we do not believe such time would have enhanced the applicability assessments. Finally, these tools were only applied by academic researchers, not by decision-makers. Decision-makers may interpret and experience these tools differently; future research could explore this. Additionally, future research should also examine which methods are best for exploring how interventions work and how such understandings could be used to make assessments of applicability. A final point that is beyond the scope of this paper is how information on contextual factors is identified and incorporated with information on mechanisms of action.

## Conclusions

Tools with ready-to-use criteria for assessing applicability are either unusable or not useful and are not the best method for assessments of the applicability of public health interventions without an understanding of their mechanisms of action. New tools continue to be developed, yet seem to be rarely used. It is time that we move on from creating more and more new tools, without reflecting on their utility. We propose a different approach to applicability assessment, focusing on mechanisms of action rather than characteristics. New approaches to assessing applicability that move away from checklist-style assessments need to be developed, tested, reported and discussed.

## Additional file

Additional file 1: Example of a completed applicability assessment pro-forma. (DOCX 41 kb)
