# Supplementary material for: Assessing the applicability of public health intervention evaluations from one setting to another: a methodological study of the usability and usefulness of assessment tools and frameworks
Source: Health Res Policy Syst. 2018 Sep 4;16:88. doi: 10.1186/s12961-018-0364-3 (PMC6122596; doi:10.1186/s12961-018-0364-3)
Supplement: Supplementary file 1 — Example of a completed applicability assessment pro-forma. (DOCX 41 kb) [file 12961_2018_364_MOESM1_ESM.docx]

**Additional file 1: Applicability assessment tool testing**

Tool/main reference: Bonell 2006

Linked papers: None

Tester (LB/DK/HB): LB

| **Criteria** | **Application to Bertz** | **Possible application to English context?** | **Summary judgment on applicability to English context** | **Comment** |
| --- | --- | --- | --- | --- |
| **Can the intervention be delivered elsewhere? - Feasibility** | *(Capacity to deliver an intervention)*  **Local champions:** No info  **Use of other systems:**   - Recruitment probably in antenatal clinics. No more info. - Paid one-year maternity leave = time and income for participants.   **Transport:** Urban area. No information on transportation time, parking and costs.  **Costs:** Costs were 302.5 USD higher per participant compared to the control group (Table 1). How can the control group have costs for PA?  **Material resources**   - Free electronic body-scale to all participants - Comprehensive booklets designed for the intervention - Softwares: Dietist XP (version 3.2, 2012, Kost och Näringsdata), the Swedish 2010 dietary database, and data from food manufacturers. - SMS   **Human resources**   - Dietitian for each participant: 1,5 h consultation at the clinic, 1h consultation at home, bi-weekly follow-ups by SMS/email, revision of 3x four-days dietary records, production of a report, and follow-up by phone or email after 6 mo on health status and research participation. - Research staff and nurses per participant: explanation and analysis of the 3x four-days dietary records, and 3x baseline measures. - Comment: The last baseline measures and dietary records were for research purposes, but I wouldn’t be surprised that they played a role on motivation. - Workload related to the dietary records: “If records were incomplete, the woman was contacted and asked to provide additional information. If such information was not available, standard servings from the dietary database were used for recorded foods. All entered and calculated dietary data were reviewed for accuracy and consistency by one dietitian” = +++ time and expertise. - No info provided on providers’ training, recruitment, level of expertise, views and links with other staff.   **Dietary intervention approach**   - Aimed to reduce dietary intake by 500 kcal/day. Goal: Reduce weight by 0.5 kg / week (but not exceed 1 kg week) to a total of 6 kg weight loss. Comment: What if some women needed to lose < 6 kg (already BMI of 25-26?) - 4 key dietary principles: limit sweets, snacks, desserts, and sugar-sweetened beverages to 100 g/wk, to be eaten during only 1 day of the week; substitute regular foods with low-fat and low-sugar alternatives; gradually increase vegetables so that vegetables cover half the plate at lunch and dinner; and reduce portion sizes by reducing equally from the major carbohydrate- and fat containing foods of the meal. If a principle did not produce the weight-loss goal during 2 weeks (0.5þ0.5 kg), the next principle in order was to be introduced. - The women were advised to introduce four key dietary principles, one at a time, at a pace that facilitated the weekly weight-loss goal, and to devote no longer than 4 weeks to a single step before introducing the next, so that the first three steps could be supported during the treatment. | **Local champions:** No info  **Use of other systems:**   - Need a referral system with other clinics and trained staff - Maternity leave shorter and often less paid.   **Transport:**   - Parking and/or public transport available? - Accessible to rural populations?   **Costs:** The cost analysis is not likely to be transferable.  **Material resources**   - Is it possible to lend scales? - To adapt and produce the booklets? - To have access to a software to analyse dietary records? - To have a mobile to send SMS to participants?   **Human resources**   - RD: probably ok to have a total of 2,5 h per participant. Are house visits, bi-weekly follow-up and validation of 3x 4-days dietary records possible? (+++ time and car). - Who would take the baseline measurements? - Who would explain and analyse the dietary records (requires +++ time and training). - Would the volume of patients be sufficient to have a dietitian?   **Dietary approach:** Probably in line with UK and RD’s recommendations. | Intervention approach plausible. The rest depends on the referral system, the role that the maternity leave played, transport, human resources (especially regarding the dietary records and biweekly follow-ups), and material resources (scale, booklets and software). | Very vague question. Some components are hidden in Table 2 (recruitment, links and views). |
| **Can the intervention be delivered elsewhere? - Coverage** | *(Reach)*   - No info on why the population is more educated, healthy etc than the average. It could be a “reach” issue. - Urban area. No information on transportation time, parking and costs. - Few visits in person needed: 4 at the clinic (3 baseline measurements + 1,5 h with the dietician), and 1h with the dietician at home. - Biweekly follow-up by phone or email. - Recruitment seems to have been done in the 15 antenatal care clinics. | - Is the clinic accessible: transport time, costs, parking, opening times? Is the population likely to go? - Are house visits possible? - The biweekly follow-up should be practical for both rural and urban areas since people don’t need to move and most people should have at least a regular phone. - A referral system would need to be thought to reach pregnant women in a range of settings and socioeconomic status. | - Depends on the accessibility of the clinic, the “reach” through the referral system and the possibility to do house visits. | Not clear. I think they mean “Reach”. Again, some components are hidden in Table 2 (social norms… and why isn’t it in “acceptability”?) |
| **Can the intervention be delivered elsewhere? - Acceptability** | - No process evaluation apart from participants’ views - No information on refusals - Good participation rate, so probably acceptable for participants: 62 women (91%) completed the intervention period, and 57 women (84%) remained to complete the 1-y follow-up - Contrary to the other groups, the reasons to drop out were not associated with BMI and N children, so more likely to be acceptable for the latter. - The dietary approach (4 principles, progressive, information provided) was a success factor of the intervention. - Having a dietitian helped for credibility. | - The intervention should be perceived as credible if a Dietitian is used in the UK. No idea if it is someone else. - The dietary approach could be accepted if well explained and offered in similar conditions. Hard to tell! | Hard to tell, but no major reason to think it wouldn’t be acceptable if offered in similar conditions. | Clearer than the other questions (also narrower). Overlaps with coverage (e.g. social norms) |
| **Recipients’ needs** | **Sociodemographic profile**   - Age: 33.7 +- 4.2 - Sex not relevant - 97% white. - Highly educated (73% of women had 3 y of education beyond high school) - Full paid 1-year maternity leave). 100% ? - 100% married/cohabitating (support) - No info on the representativeness of participants to the target population (likely to be very low).   **Health needs and habits**   - All plan to breastfeed for >= 6 mo - All non-smoking - All physically active throughout the study (8000 steps/d, w75 min/d). - No ill babies and mothers - BMI 25-35 - Baseline intake of fat and sucrose above, and an intake of total carbohydrates and fiber below, recommended levels. - Small proportion met the recommended intake for vitamin D (15%), folate (13%), and iron (30%), but 72% met the recommended intake for calcium. - “20% of infant energy intake as complementary foods” how can they know?,   **Attitude and knowledge**   - Seem motivated (already physically active, plan to breastfeed > 6 mo) - Most were motivated to weight loss and considered themselves well informed regarding official diet and exercise recommendations. - “Before joining, the women perceived themselves as lacking lifestyle control, self-control and the ability to maintain a healthy weight”. - Their major barriers to weight loss/maintenance were stress, social norms, lack of structure or discipline, and lack of sufficient motivation. These were also barriers to lifestyle change during the intervention.   **Causal pathway** presented. | **Sociodemographic profile**   - UK average younger, less educated, more ethnic and probably lower income (or some no income). - Less likely to have support.   **Health needs and habits**   - Breastfeed rates lower, and for a shorter time - Less healthy habits (smoking and PA) - Dietary intake of macronutrients might be similar. Not sure for micronutrients (Ca could play a role in weight loss). - Less healthy (no illness was included, so no Db, HTA, CDV, etc, which are frequent among obese people). - Would mothers of low-weight babies and ill babies be included? - Would people with a BMI of 25-26 be accepted? - Would BMI > 35 be excluded?   **Attitude and knowledge**   - Less informed than study group. - No idea for motivation level. - Likely to face the same barriers.   **Causal pathway** has potential to work in the UK. Key factors have been identified. | Population: Impact likely to be smaller in the UK (if the general population is included).  Knowledge and motivation potentially lower in the UK. Would need adjustments (especially that the population is less educated, doesn’t all have access to Internet and have less support).  Causal pathway: Potential to be generalizable to the UK. | This is a very hectic category! Very relevant, but should be split in different questions. |

**Reflections**

| General impressions | 1. The questions are too vague and broad:   - One question could be divided in multiple questions with indicators to better guide the user. To overcome this, I have created categories within some questions.   *E.g.1: “Coverage: ways of reaching the population, including issues in the health system and in rural settings”.*  *E.g.2: “Recipients’ needs” includes socio-demographic characteristics, health needs, attitude and knowledge, and causal pathways.*   - We need to first think about the factors that are important to answer the question. - We are likely to miss the important information, especially the one that is NOT reported. - I feel that some answers fit in more than one question (e.g. social norms, transport). - Results are likely to vary according to the user.   2. Not ready-to-be-used:   - Some elements are listed in the text while others are in Table 2. - The elements in table 2 were for a specific example. I guess other concepts would have been highlighted for another topic. |
| --- | --- |
| Any particularly useful criteria? | - Feasibility: - Some interventions require the existence of other health services (so reliance on other services) – Not included in Cambon - Views of providers - Costs (but subjective. I have divided them into material and human resources) - Coverage: representativeness of participants to the target population - Needs: - Causal pathway - Sociodemographic characteristics (but no guideline) |
| Any particularly unhelpful criteria? Not relevant |  |
| Any particularly unhelpful criteria? Not realistic | Feasibility – local champions: rarely reported. |
| Any criteria/questions that were missing? (that we think should’ve been considered) | - Participants’ motivation (like in Cambon) - Providers’ attitude and their impact on the intervention |
| Other thoughts |  |
| Overall useabilty of tool | Difficult, but actually, it’s more a commentary than a tool. |
| Time taken to complete | 3,5 hours |
| General impressions of generalisability to English context | - Dietary approach and causal pathway suitable to the UK. - However, seems very demanding in HR, the population not representative of the UK, and the maternity leave seems to have played a role but is shorter in the UK. - If the budget is there, the programme and material are adapted for less educated and healthy people, the referral system allows to reach a great proportion of the population, the clinic is accessible, it is possible to do home visits, and participants are incited to walk +++, *maybe* it could be generalisable… So in practice, I guess that chances are small. |
